# Prostate secretory protein 94 inhibits sterol binding and export by the mammalian CAP protein CRISP2 in a calcium-sensitive manner

**DOI:** 10.1016/j.jbc.2022.101600

**Published:** 2022-01-19

**Authors:** Ola El Atab, Aslihan Ekim Kocabey, Oluwatoyin A. Asojo, Roger Schneiter

**Affiliations:** 1 Department of Biology, University of Fribourg, Fribourg, Switzerland; 2 Department of Chemistry and Biochemistry, Hampton University, Hampton, Virginia, USA

**Keywords:** CAP protein superfamily, PSP94, sterol binding, CRISP, antifungal activity, CBM, caveolin binding motif, CRD, cysteine-rich domain, CRISP, cysteine-rich secretory protein, FAME, fatty acid methyl ester, HA, hemagglutinin, MST, microscale thermophoresis, PR-1, pathogenesis-related protein 1, Pry, pathogen related in yeast, PSP94, prostate secretory protein of 94 amino acid, SC, synthetic complete, TCA, trichloroacetic acid

## Abstract

Members of the CAP protein superfamily are present in all kingdoms of life and have been implicated in many different processes, including pathogen defense, immune evasion, sperm maturation, and cancer progression. Most CAP proteins are secreted glycoproteins and share a unique conserved αβα sandwich fold. The precise mode of action of this class of proteins, however, has remained elusive. *Saccharomyces cerevisiae* has three CAP family members, termed pathogen related in yeast (Pry). We have previously shown that Pry1 and Pry2 export sterols *in vivo* and that they bind sterols *in vitro*. This sterol binding and export function of yeast Pry proteins is conserved in the mammalian CRISP proteins and other CAP superfamily members. CRISP3 is an abundant protein of the human seminal plasma and interacts with prostate secretory protein of 94 amino acids (PSP94), another major protein component in the seminal plasma. Here we examine whether the interaction between CRISP proteins and PSP94 affects the sterol binding function of CAP family members. We show that coexpression of PSP94 with CAP proteins in yeast abolished their sterol export function and the interaction between PSP94 and CAP proteins inhibits sterol binding *in vitro*. In addition, mutations that affect the formation of the PSP94–CRISP2 heteromeric complex restore sterol binding. Of interest, we found the interaction of PSP94 with CRISP2 is sensitive to high calcium concentrations. The observation that PSP94 modulates the sterol binding function of CRISP2 in a calcium-dependent manner has potential implications for the role of PSP94 and CRISP2 in prostate physiology and progression of prostate cancer.

CAP proteins form a large superfamily with members found in all kingdoms of life. The superfamily was named after the identification of sequence similarities between its founding members: CRISPs (cysteine-rich secretory proteins) of the mammalian reproductive tract, Ag5 (antigen 5) in the venom secretory duct of stinging insects, and PR-1 (pathogenesis-related protein 1), which is induced in plants upon pathogen infection. They are also referred to as sperm coating proteins (SCPs) or TAPS (Tpx-1/Ag5/PR-1/Sc7). CAP proteins share a unique conserved CAP domain, which adopts a three-layered αβα sandwich fold with a central three-stranded antiparallel β-sheet flanked by α-helices on both sides (1). They are mostly secreted glycoproteins, implicated in many fundamental biological processes including immune defense in mammals and plants, sperm maturation and fertilization, prostate and brain cancer, pathogen virulence, and venom toxicity. Even though these proteins are extensively studied, their precise mode of action remains to be defined (2, 3, 4).

Yeast cells express three CAP family members, pathogen related in yeast (Pry) 1–3. Pry1 and Pry2 are secreted glycoproteins, whereas Pry3 is a glycosylphosphatidyl inositol–anchored cell wall protein. Mutant cells lacking all three Pry proteins are viable, but they fail to secrete acetylated sterols. This block in sterol secretion is rescued by expression of mammalian CAP family members, CRISP2, and purified Pry1, Pry2, and CRISP2 bind radiolabeled cholesterol in the low micromolar range *in vitro* (5). The sterol binding function of the CAP domain was mapped to a flexible hydrophobic loop, known as the caveolin binding motif (CBM). Mutations within this motif abrogate sterol binding, suggesting that CAP proteins can bind sterols through displacement of this hydrophobic loop (6, 7, 8). These results indicate that sterol export is a conserved feature of CAP superfamily members. Consistent with this proposition, plant PR-1 proteins bind sterols *in vitro* and inhibit growth of sterol auxotrophic pathogenic oomycetes (9).

The mammalian CRISP proteins are composed of two domains: an N-terminal CAP domain and a C-terminal cysteine-rich domain (CRD) also known as ion channel regulatory domain (ICR), connected by a hinge region (10, 11, 12). These proteins are present in the mammalian reproductive tract and in the venom secretory ducts of reptiles (2, 13). Within the male reproductive tract, CRISPs associate with the sperm surface and are required for epididymal sperm maturation and motility. They also function in the female reproductive tract, where they are important for sperm chemotaxis and efficient fertilization (12, 14). Transcriptional deregulation of some CRISP proteins in malignant cells suggests a possible function of these proteins in cancer development or progression. For example, human CRISP3 is dramatically upregulated in prostate cancer and serves as diagnostic and prognostic marker of prostate cancer progression (15).

In the seminal plasma, CRISP3 interacts with prostate secretory protein of 94 amino acids (PSP94), also known as β-microseminoprotein (MSP/MSMB), a member of the immunoglobulin binding factor family. PSP94 is one of the most abundant proteins secreted by the prostate gland, but it is also present in other body fluids, including the tracheobronchial fluid (16, 17, 18). It was identified as an inhibitor of sperm motility, but its precise function in prostate physiology has yet to be defined (19, 20). In contrast to CRISP3, PSP94 expression is downregulated in prostate cancer, suggesting a protective role in prostate carcinogenesis (21, 22, 23). PSP94 also interacts with another CRISP family member, CRISP2, also referred to as Tpx-1 (testis-specific protein 1), a nonglycosylated CRISP family member produced during spermatogenesis, which shares 71% sequence identity with CRISP3 (24, 25). CRISP2 regulates calcium influx through ryanodine receptors to modulate sperm flagellar motility (11, 26, 27). Its expression is thus associated with human spermatogenesis and infertility (28).

In this study, we aimed to characterize the functional significance of the interaction between PSP94 and CAP proteins in general and that of the CRISP family members, CRISP2 in particular. Coexpression of PSP94 with either CRISP2 or CRISP3 in yeast cells blocks the sterol export function of CRISP proteins *in vivo* but not the secretion of these proteins. PSP94 and CRISP2 are secreted as heteromeric protein complex as revealed by coimmunoprecipitation. Purified PSP94 binds CRISP2 and CRISP3 with nanomolar affinity and inhibits sterol binding of CRISP2 *in vitro*. Inhibition of this sterol binding function of CRISP2 by PSP94 is specific and does not affect the fatty acid binding and export function of CAP proteins or that of CRISP2. Mutations that block the interaction between PSP94 and CRISP2, on the other hand, restore sterol export by CAP proteins *in vivo* and sterol binding *in vitro*. Remarkably, this interaction between PSP94 and CRISP2 is sensitive to high calcium concentrations, allowing CRISP2 to bind sterols even in the presence of PSP94. Potential implications of the calcium-dependent interaction between PSP94 and CRISP2 on prostate physiology and their potential role in prostate cancer progression are discussed.

## Results

### Expression of PSP94 blocks sterol export but not secretion of CRISP proteins

**Figure 1:**
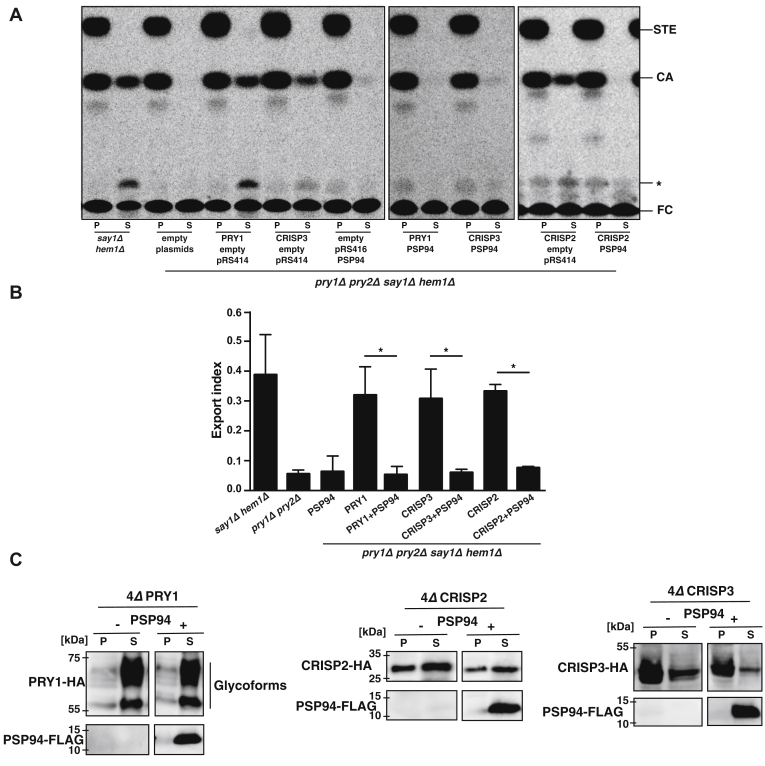
**PSP94 inhibits sterol export by CAP proteins.***A*, export of acetylated cholesterol is blocked in cells expressing PSP94. Acetylation and export of [^14^C]cholesterol were examined in *hem1Δ say1Δ* double mutant cells and in quadruple mutant cells lacking the endogenous CAP family members Pry1 and Pry2 (*pry1Δ pry2Δ hem1Δ say1Δ*). Strains expressing the indicated CAP protein from a plasmid (PRY1, CRISP2, or CRISP3) and coexpressing either PSP94 or an empty control plasmid, pRS414, were cultivated in the presence of [^14^C]cholesterol. Lipids were extracted from cell pellet (P) and culture supernatant (S), separated by TLC, and visualized by phosphorimaging. The position of free cholesterol (FC), cholesteryl acetate (CA), steryl esters (STE), and an unidentified lipid (∗) are indicated to the *right*. *B*, quantification of cholesteryl acetate export. The plotted export index indicates the relative levels of cholesteryl acetate exported by the cells. The export index corresponds to the ratio of extracellular cholesteryl acetate to the sum of intracellular and extracellular cholesteryl acetate. Data correspond to means ± SD of three independent experiments, and statistical significance is indicated: ∗*p* ≤ 0.05. *C*, expression of PSP94 does not block the synthesis or secretion of CAP family members. Proteins were precipitated using trichloroacetic acid from the culture medium (S) and cells (P) expressing HA-tagged Pry1, CRISP2, or CRISP3 in the presence or absence of FLAG-tagged PSP94 and analyzed by Western blotting.

The yeast CAP protein Pry1 has previously been shown to bind and export cholesteryl acetate (5). To test whether expression of PSP94 would affect the function of Pry1 in sterol export, we expressed PSP94 fused to the N-terminal signal sequence from Pry1 (amino acids 1–19) from a constitutive alcohol dehydrogenase promoter (*ADH1*) to direct the protein into the yeast secretory pathway. This PSP94-containing plasmid was then transformed into heme-deficient yeast cells lacking the sterol deacetylase Say1 (*hem1Δ say1Δ*). This strain is able to take up radiolabeled [^14^C]cholesterol, acetylate it in an alcohol acetyltransferase 2 (Atf2)-dependent pathway, and secrete the resulting radiolabeled cholesteryl acetate back into the culture medium (29). Secretion of cholesteryl acetate requires the yeast CAP family members Pry1 and Pry2 and is blocked in a *hem1Δ say1Δ pry1Δ pry2Δ* quadruple mutant (5). The block in sterol secretion could be relieved by expression of Pry1 or the mammalian CAP family member CRISP2 and CRISP3 (5) (Fig. 1*A*). Co-expression of PSP94 with Pry1, CRISP2, or CRISP3, however, blocked sterol secretion by 78% to 82% (Fig. 1, *A* and *B*). To test whether expression of PSP94 would affect the synthesis or secretion of Pry1, CRISP2, or CRISP3, we coexpressed FLAG-tagged PSP94 with hemagglutinin (HA)-tagged Pry1, CRISP2, or CRISP3, separated yeast cells from the culture medium, precipitated proteins from both fractions with trichloroacetic acid (TCA), and detected the tagged proteins by Western blotting. The HA-tagged CAP family members were present in both the cells and the culture medium, whereas FLAG-tagged PSP94 was mostly present in the medium, indicating that the protein is efficiently secreted (Fig. 1*C*). These results indicate that PSP94 does hamper sterol export mediated by Pry1, CRISP2, or CRISP3 not by disturbing their synthesis and/or secretion but by interfering with the sterol binding function of these CAP proteins.

We and others have previously shown that CAP proteins can bind eicosanoids and free fatty acids at a second lipid-binding site that is independent of the sterol-binding site (30, 31). To test whether PSP94 affects fatty acid binding by the CAP domain, we examined whether coexpression of PSP94 with CRISP2 affects fatty acid export from mutant cells lacking the two acyl-CoA synthases Faa1 and Faa4 and two of the three yeast CAP domain proteins, Pry1 and Pry3. Cells coexpressing PSP94 and CRISP2, however, displayed similar levels of fatty acid export as cells expressing only Pry1 or CRISP2, indicating that PSP94 does not affect fatty acid binding and export by CAP family members (Fig. S1). This result suggests that PSP94 specifically affects the sterol-binding site of the CAP domain but not the eicosanoid/fatty acid–binding pocket.

### PSP94 binds CAP proteins *in vitro* and *in vivo*

**Figure 2:**
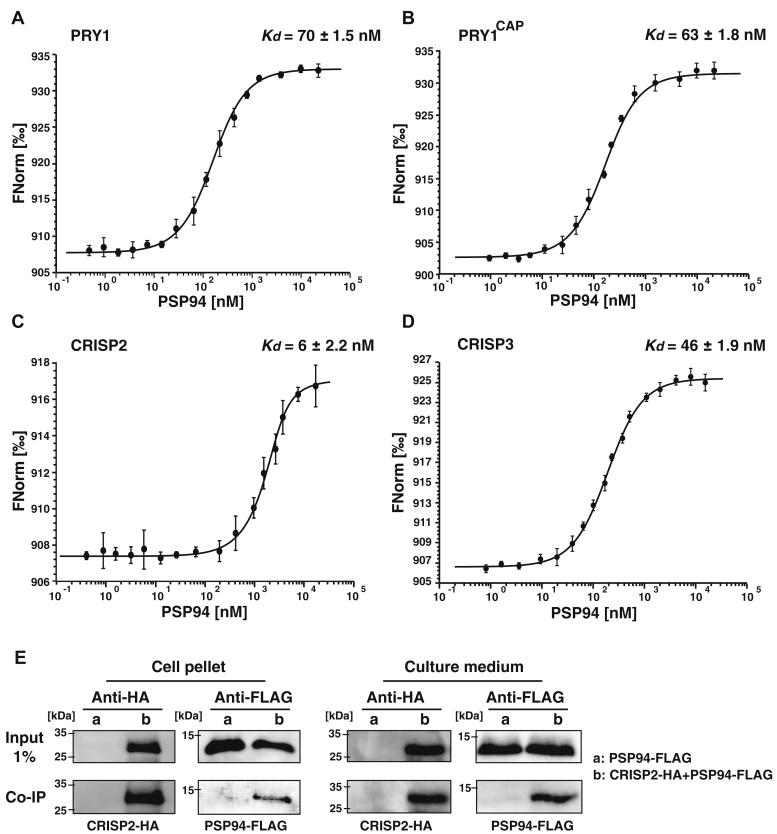
**PSP94 binds CAP proteins *in vitro***. *A*–*D*, the interaction between PSP94 and Pry1, the CAP domain of Pry1 (Pry1^CAP^), CRISP2, or CRISP3 was assessed by microscale thermophoresis. Purified and fluorescently labeled CAP proteins were incubated with increasing concentrations of unlabeled PSP94 and complex formation was analyzed. Measurements were performed in triplicates, and the corresponding dissociation constants (*K*_*d*_) are indicated. *E*, PSP94 coimmunoprecipitates with CRISP2. Cells expressing FLAG-tagged PSP94 (a) and cells coexpressing FLAG-tagged PSP94 together with HA-tagged CRISP2 (b) were cultivated, and the interaction between PSP94 and CRISP2 in the cell lysate (pellet) and the culture medium (supernatant) was analyzed by immunoprecipitation with an anti-HA antibody followed by Western blotting to detect both HA-tagged CRISP2 and FLAG-tagged PSP94.

To confirm the direct interaction of PSP94 with CRISP3 and to test whether PSP94 would also bind other CAP family members such as yeast Pry1, or CRISP2, we performed *in vitro* protein binding assay using microscale thermophoresis (MST). Therefore, PSP94 and the CAP family members, Pry1, CRISP2, and CRISP3, were expressed as fusion proteins with a C-terminal polyhistidine tag in *Escherichia coli*, and the proteins were affinity purified on nickel-NTA agarose beads. The purified CAP proteins were then fluorescently labeled using RED-tris-NTA and incubated with varying concentrations of PSP94. The concentration-dependent interaction of PSP94 with the fluorescently labeled CAP proteins was then monitored by MST and the resulting binding curves are shown in Figure 2, *A*–*D*. These results indicate that PSP94 binds CAP family members with nanomolar affinity, ranging from 6 to 70 nM for binding of CRISP2 and Pry1, respectively. PSP94 thus has high binding affinity for all three CAP proteins tested, and it also binds the CAP domain of Pry1 (Pry1^CAP^) alone. This indicates that PSP94 recognizes a binding site that is shared among different CAP family members and that is present within the CAP domain of these proteins, but not in their C-terminal extension, *i.e.*, the hinge region or the CRD/ICR of CRISP2 and CRISP3.

To test whether PSP94 also interacts with CAP family members when expressed in yeast cells, we generated strains coexpressing FLAG-tagged PSP94 with HA-tagged CRISP2. We then immunoprecipitated CRISP2-HA either from the cell pellet or the culture medium and tested whether PSP94-FLAG was coprecipitated (Fig. 2*E*). Coprecipitation of PSP94-FLAG with CRISP2-HA was observed both in protein extracts from the cell pellet and the culture medium, suggesting that these proteins interact with each other already in the lumen of the secretory pathway and then stay in a heteromeric complex once they are secreted into the culture medium. This coprecipitation was specific as PSP94-FLAG was not detected in cells that did not express CRISP2-HA.

### PSP94 inhibits sterol binding of CAP proteins

**Figure 3:**
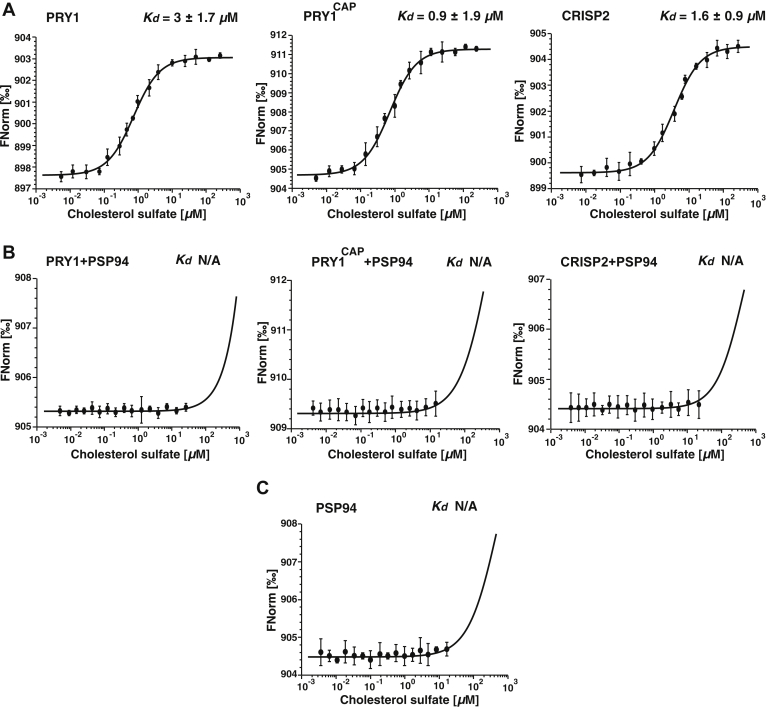
**PSP94 inhibits sterol binding by CAP proteins.***A*–*C*, sterol binding by CAP family members in the absence (*A*) or presence of PSP94 (*B*) was assessed by microscale thermophoresis. CAP family members bound cholesterol sulfate in the micromolar range, but this binding is blocked in the presence of PSP94, which by itself does not bind cholesterol sulfate (*C*). Measurements were performed in triplicates, and the corresponding dissociation constants (*K*_*d*_) are indicated. N/A, not applicable.

To test whether the interaction between PSP94 and CAP family members affects their sterol binding *in vitro*, we performed ligand binding assays using a more water-soluble cholesterol derivative, cholesterol sulfate. The CAP family members all bound cholesterol sulfate in the micromolar range, whereas PSP94 did not bind this cholesterol derivative as determined by MST (Fig. 3, *A* and *C*). In the presence of an equimolar concentration of PSP94, however, sterol binding by the CAP proteins was blocked (Fig. 3*B*). These results show that complex formation between CAP proteins and PSP94 affects sterol binding by CAP proteins, suggesting that PSP94 could act as a modulator of CAP proteins under physiological conditions.

### Mutations within CRISP2 that abolish the interaction with PSP94 restore sterol binding in the presence of PSP94

**Figure 4:**
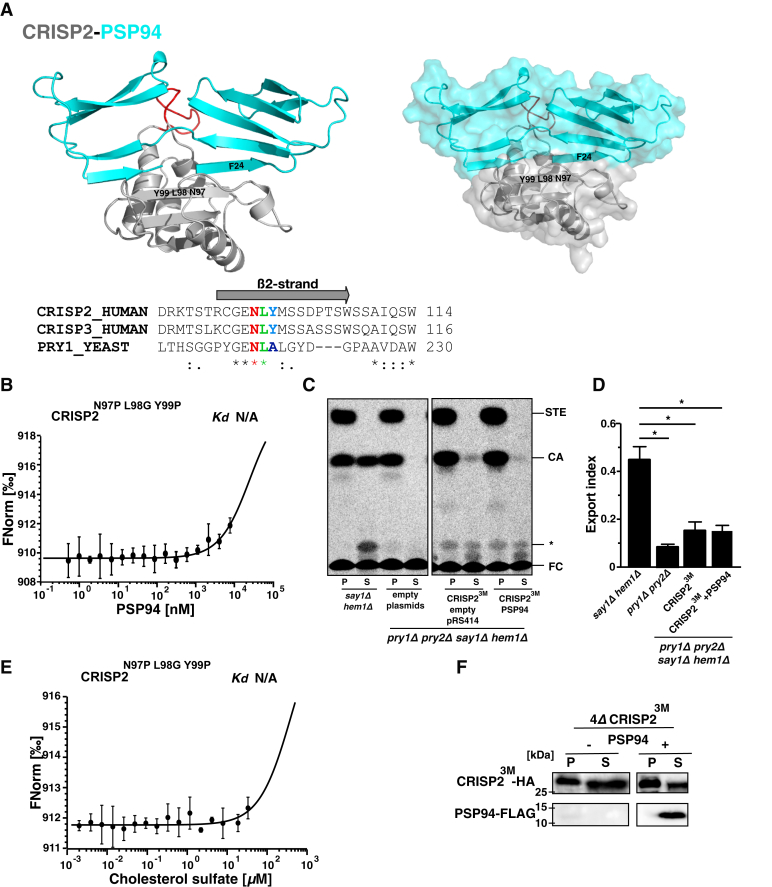
**Conserved residues within the β2-strand of CRISP2 are crucial for the interaction with PSP94 and also affect sterol binding by the CAP domain.***A*, models of the CRISP2–PSP94 complex. The structure of CRISP2 is shown in *gray* with the caveolin binding motif indicated in *red*. PSP94 is shown in *blue*. Residues that are important for the interaction between CRISP2 (N97 and L98 in the β2-strand) and PSP94 (F24 in the N terminus) are indicated: N97, L98, and Y99 in the β2-strand of CRISP2 and F24 in the N-terminal part of PSP94. Conservation of N97 and L98 is shown in the sequence alignment of CRISP2, CRISP3, and Pry1. The *gray arrow* indicates the extent of the β2-strand. *B*, the triple mutant version of CRISP2, CRISP2^N97P L98G Y99P^, does not interact with PSP94. CRISP2^N97P L98G Y99P^ was expressed and purified from *E. coli*, and the interaction between the triple mutant and PSP94 was assessed *in vitro* by microscale thermophoresis. Measurements were performed in triplicates. No corresponding dissociation constant (*K*_*d*_) could be deduced. *C*, the mutant version of CRISP2^N97P L98G Y99P^ (CRISP2^3M^) does not export cholesterol *in vivo*. Quadruple mutant cells (*pry1Δ pry2Δ say1Δ hem1Δ*) expressing either empty plasmids or plasmids containing CRISP2^3M^ with or without PSP94 were labeled with [^14^C]cholesterol. Lipids were extracted from the cell pellet (P) and the culture supernatant (S), separated by TLC, and quantified. *D*, relative export of radiolabeled cholesteryl acetate is plotted as export index in the graph. Data correspond to means ± SD of three independent determinations, and statistical significance is indicated: ∗*p* ≤ 0.05. *E*, the triple mutant version of CRISP2 does not bind cholesterol sulfate. Purified CRISP2^N97P L98G Y99P^ was incubated with increasing concentrations of cholesterol sulfate *in vitro* and lipid binding was assessed by microscale thermophoresis. Measurements were performed in triplicates. No corresponding dissociation constant (*K*_*d*_) could be deduced. *F*, the triple mutant version of CRISP2 is expressed and secreted. Quadruple mutant cells (*pry1Δ pry2Δ say1Δ hem1Δ*) expressing a HA-tagged triple mutant version of CRISP2 (CRISP2^3M^) either with or without a FLAG-tagged version of PSP94 were cultivated. Proteins from both the cell pellet (P) and culture supernatant (S) were TCA precipitated and detected by Western blotting. N/A, not applicable.

Previous structural characterization of PSP94–CRISP3 interaction has suggested that PSP94 binds CRISP3 through antiparallel beta-sheet interactions between the N-terminal β1-strand of PSP94 and the accessible β2-strand of CRISP3, thereby disrupting homodimer formation of PSP94 (32, 33). This interaction is thus located outside the hydrophobic loop that harbors the sterol-binding CBM. Based on these structural considerations, it is thus not apparent why and how the interaction between PSP94 and CRISP2 would block sterol binding at the CBM. To investigate this question in more detail, we first performed computational docking studies to identify residues in CRISP2 that are critical for the interaction with PSP94. These studies indicated that binding of PSP94 to CRISP2 indeed covers the CBM and that three residues in β2-strand of CRISP2, N97, L98, and Y99, are critical for the interaction with PSP94 (Fig. 4*A*). Two of these three interacting residues, N97 and L98, are conserved in all three CAP family members tested here, CRISP2, CRISP3, and Pry1 (Fig. 4*A*). To identify mutations that would only affect the interaction of CRISP2 with PSP94, but not the capacity of CRISP2 to bind sterols, we first generated mutant versions of CRISP2 in which each one of the three interacting amino acids was mutated individually: N97P, L98G, and Y99P. Mutations to proline were chosen with the aim of disrupting the antiparallel β-sheet interactions between the β2-strand of CRISP2 and the β1-strand of PSP94. All three of these single-mutant versions of CRISP2 could still bind cholesterol sulfate *in vitro*, and this sterol binding capacity was also impaired in the presence of PSP94, indicating that the single point mutant versions of CRISP2 still interacted with PSP94, as was confirmed by MST (Fig. S2, *A*–*C*). However, in a mutant version of CRISP2, which bears all three mutations simultaneously, CRISP2^N97P L98G Y99P^, the interaction with PSP94 was indeed abolished (Fig. 4*B*). Unfortunately, this triple mutant version of CRISP2 was also impaired in sterol binding *in vitro* and sterol export *in vivo*, indicating that the residues that are important for interaction with PSP94 are also important for sterol binding by the protein (Fig. 4, *C*–*E*). A HA-tagged version of the CRISP2^N97P L98G Y99P^ triple mutant was stably produced when expressed in yeast and was secreted, as was FLAG-tagged PSP94 when coexpressed with it (Fig. 4*F*). Thus, the defect in sterol export of the triple mutant CRISP2 is not due to its instability or lack of secretion.

**Figure 5:**
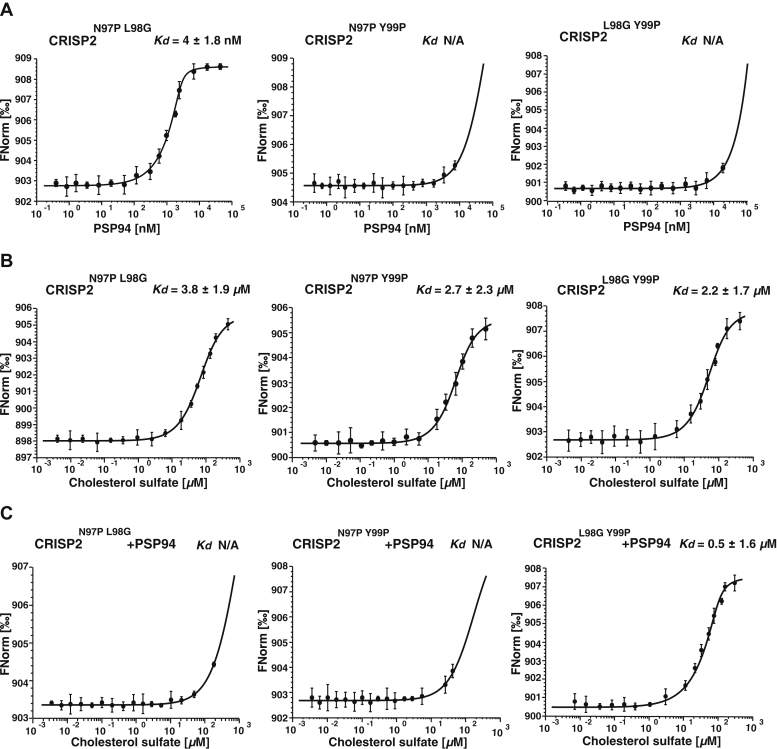
**Identification of two key residues within CRISP2 that block the interaction with PSP94 but not binding of cholesterol.***A*–*C*, all three double mutant combinations of CRISP2 in the three residues that are predicted to interact with PSP94, N97, L98, and Y99 were generated. The mutant proteins were expressed in *E. coli* and purified. Their binding to PSP94 (*A*), cholesterol sulfate (*B*), and cholesterol sulfate in the presence of PSP94 (*C*) was assessed by microscale thermophoresis. The CRISP2^L98G Y99P^ double mutant is the sole of the three double mutant versions that retained the capacity to bind cholesterol sulfate even in the presence of PSP94. Measurements were performed in triplicates, and the corresponding dissociation constants (*K*_*d*_) are indicated. N/A, not applicable.

**Figure 6:**
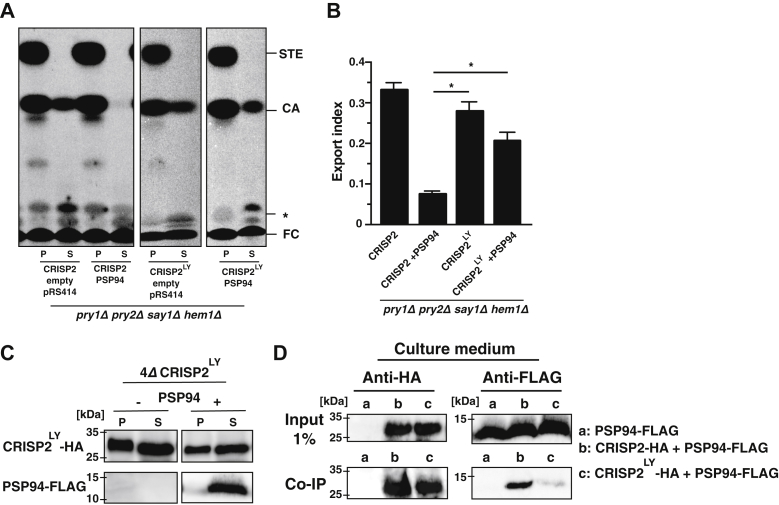
**The CRISP2 double mutant version CRISP2**^**L98G Y99P**^**binds and exports sterols *in vivo* even in the presence of PSP94.***A*, sterol export by CRISP2^L98G Y99P^ in the presence of PSP94. Quadruple mutant cells (*pry1Δ pry2Δ say1Δ hem1Δ*) expressing either empty plasmids or plasmids containing the double mutant version of CRISP2 (CRISP2^LY^) with or without PSP94 were labeled with [^14^C]cholesterol. Lipids were extracted from both the cell pellet (P) and the culture supernatant (S), separated by TLC, and quantified. *B*, export of radiolabeled cholesteryl acetate is plotted as export index in the graph. Data correspond to means ± SD of three independent determinations, and statistical significance is indicated: ∗*p* ≤ 0.05. *C*, the double mutant version of CRISP2 (CRISP2^LY^) is expressed and secreted in the absence or presence of PSP94. Cells expressing an HA-tagged double mutant version of CRISP2 (CRISP2^LY^) either with or without a FLAG-tagged version of PSP94 were cultivated, and proteins from the cell pellet (P) or culture supernatant (S) were precipitated using trichloroacetic acid and detected by Western blotting. *D*, PSP94 only weakly interacts with CRISP2^LY^. Cells expressing FLAG-tagged PSP94 (a) and cells coexpressing FLAG-tagged PSP94 together with HA-tagged wildtype CRISP2 (b) or the double mutant version of CRISP2^LY^ (c) were cultivated, and the interaction between PSP94 and CRISP2 in the culture medium was analyzed by immunoprecipitation with an anti-HA antibody followed by Western blotting to detect both HA-tagged CRISP2 and FLAG-tagged PSP94.

Next, we generated all three double mutant combinations of CRISP2: N97P L98G, N97P Y99P, and L98G Y99P. These three double mutant versions of CRISP2 were again expressed as His-tagged proteins in *E. coli* and purified, and their capacity to bind sterols in the presence or absence of PSP94 was assessed by MST. This analysis revealed that the CRISP2^L98G Y99P^ double mutant was the sole of the three double mutant versions that retained the capacity to bind cholesterol sulfate in the presence of PSP94 *in vitro* (Fig. 5, *A*–*C*). The capacity of the CRISP2^L98G Y99P^ double mutant to bind sterols even in the presence of PSP94 was confirmed by the *in vivo* sterol export assay (Fig. 6, *A* and *B*). When expressed in yeast cells, the CRISP2^L98G Y99P^ double mutant was stably expressed and secreted as monitored by Western blotting (Fig. 6*C*). However, the CRISP2^L98G Y99P^ double mutant displayed strongly reduced interaction with PSP94 when a HA-tagged version of CRISP2^L98G Y99P^ was immunoprecipitated and binding of FLAG-tagged PSP94 was monitored (Fig. 6*D*). These data thus indicate that the β2-strand within the CAP domain is of critical importance for the interaction with PSP94 and its docking to PSP94 prevents sterol binding by the CAP domain.

### CRISP2 still exports cholesterol in the presence of a point mutant version of PSP94

**Figure 7:**
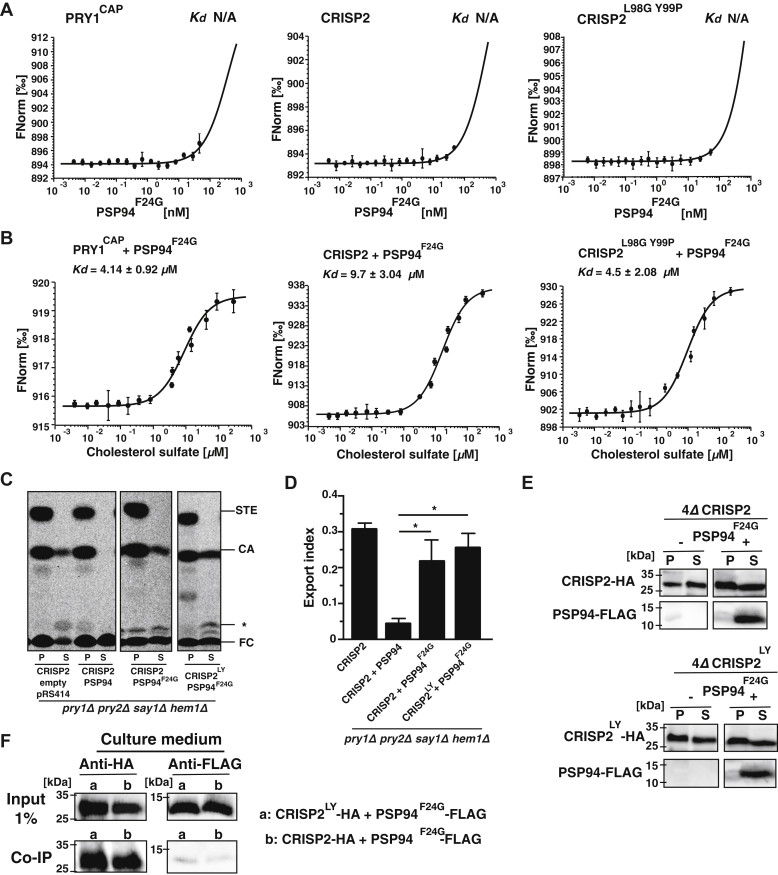
**A point mutant version of PSP94, PSP94**^**F24G**^**, fails to interact with CRISP2**. *A*, PSP94^F24G^ fails to bind the CAP domain of Pry1 (Pry1^CAP^), CRISP2, or CRISP2^L98G Y99P^. PSP94^F24G^ was expressed in *E. coli* and purified, and its interaction with Pry1^CAP^, CRISP2, or CRISP2^L98G Y99P^ was assessed by microscale thermophoresis. Measurements were performed in triplicates, and the corresponding dissociation constants (*K*_*d*_) are indicated. *B*, PSP94^F24G^ does not inhibit sterol binding by CRISP2, CRISP2^L98G Y99P^, or Pry1^CAP^. PSP94^F24G^ was preincubated with CRISP2, CRISP2^L98G Y99P^, or Pry1^CAP^ for 5 min at 24 °C, and binding of cholesterol sulfate by the indicated CAP protein was assessed by microscale thermophoresis. Measurements were performed in triplicates, and the corresponding dissociation constants (*K*_*d*_) are indicated. *C*, sterol export by CRISP2 *in vivo* is not blocked in the presence of the PSP94^F24G^ mutant version. Quadruple mutant cells (*pry1Δ pry2Δ say1Δ hem1Δ*) expressing either wildtype or the indicated mutant versions of CRISP2 (CRISP2^L98G Y99P^) and PSP94 (PSP94^F24G^) were labeled with [^14^C]cholesterol. Lipids were extracted from the cell pellet (P) and the culture supernatant (S), separated by TLC and quantified. *D*, export of radiolabeled cholesteryl acetate is plotted as export index in the graph. Data correspond to means ± SD of three independent determinations, and statistical significance is indicated: ∗*p* ≤ 0.05. *E*, the PSP94^F24G^ mutant version of PSP94 is expressed and secreted. Cells expressing HA-tagged CRISP2 or CRISP2^L98G Y99P^ either with or without a FLAG-tagged point mutant version of PSP94 (PSP94^F24G^) were cultivated, and proteins from the cell pellet (P) or culture supernatant (S) were TCA precipitated and detected by Western blotting. *F*, PSP94^F24G^ only weakly interacts with CRISP2. Cells expressing FLAG-tagged PSP94^F24G^ together with an HA-tagged CRISP2^L98G Y99P^ (a) or with wildtype CRISP2 (b) were cultivated and the interaction between PSP94 and CRISP2 in the culture medium was analyzed by immunoprecipitation with an anti-HA antibody followed by Western blotting to detect both HA-tagged proteins and FLAG-tagged PSP94^F24G^. N/A, not applicable.

To corroborate our previous results, we generated a point mutant version of PSP94, PSP94^F24G^ (F24 corresponds to F4 in the processed, signal-sequence cleaved version of PSP94), which was previously reported to have impaired binding to CRISP3 (34). PSP94^F24G^ indeed failed to bind Pry1^CAP^, CRISP2, or the double mutant CRISP2^L98G Y99P^ *in vitro* when tested by MST (Fig. 7*A*), and all three CAP proteins retained their capacity to bind sterols in the presence of this mutated form of PSP94 (Fig. 7*B*). Similarly, when tested for sterol binding and export *in vivo*, cells expressing CRISP2 exported sterols in the presence of PSP94^F24G^, indicating that the mutant version of PSP94 did not inhibit sterol binding and export by the CAP family member CRISP2 (Fig. 7*C*). Consistent with this, cells expressing PSP94^F24G^ regained an export index comparable with that of cells expressing CRISP2 alone (Fig. 7*D*). Both wildtype CRISP2 and the PSP94^F^^2^^4G^ mutant version were stably expressed and secreted from cells (Fig. 7*E*), but PSP94^F24G^ failed to coprecipitate with CRISP2 (Fig. 7*F*), confirming the lack of interaction observed by MST. When wildtype CRISP2 was replaced by the CRISP2^L98G Y99P^ mutant version, no further reduction in interaction with PSP94^F24G^ was observed either in sterol export or in protein–protein interaction (Fig. 7, *A*–*F*). Taken together, these data indicate that physical interaction between CRISP2 and PSP94 is required to block sterol binding by CRISP2 *in vivo* and *in vitro*.

### The interaction between CRISP2 and PSP94 is calcium sensitive

**Figure 8:**
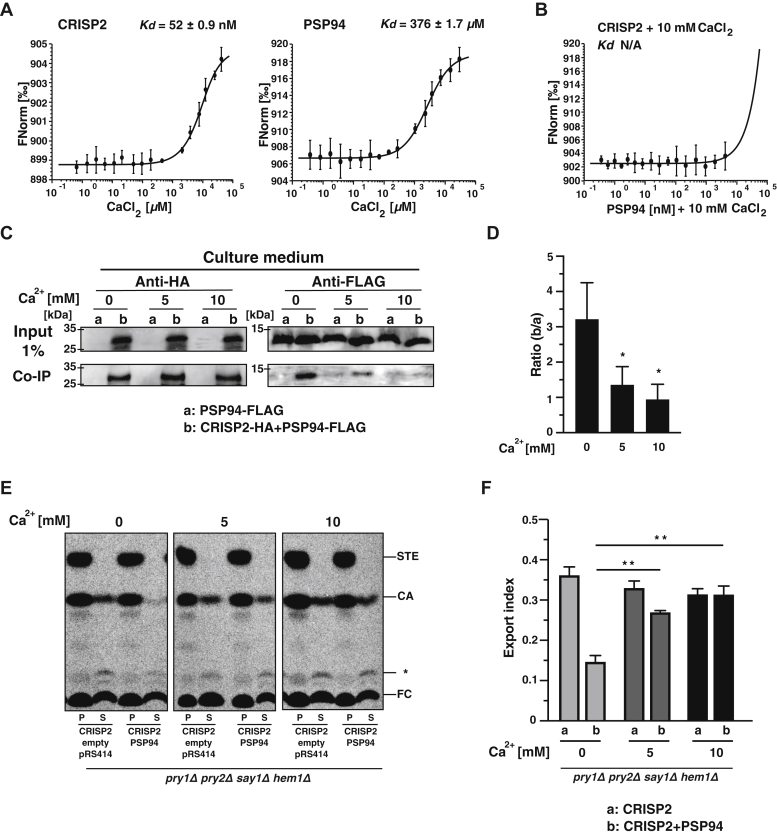
**The interaction of PSP94 with CRISP2 is sensitive to calcium**. *A* and *B*, CRISP2 and PSP94 both bind calcium *in vitro* and the interaction between CRISP2 and PSP94 is disrupted at high calcium concentrations. Calcium binding by CRISP2 and PSP94 was assessed by MST (*A*). Formation of the heteromeric complex between CRISP2 and PSP94 in the presence of 10 mM CaCl_2_ (*B*) was assessed by microscale thermophoresis. Measurements were performed in triplicates, and the corresponding dissociation constants (*K*_*d*_) are indicated. *C*, the interaction between CRISP2 and PSP94 is disrupted by calcium *in vivo*. Cells expressing FLAG-tagged PSP94 alone (a) or together with an HA-tagged CRISP2 (b) were cultivated in media containing the indicated concentration of CaCl_2_ (0, 5, 10 mM). The interaction between PSP94 and CRISP2 in the culture medium was analyzed by immunoprecipitation with an anti-HA antibody followed by Western blotting to detect both HA-tagged CRISP2 and FLAG-tagged PSP94. *D*, quantification of the interaction between CRISP2 and PSP94. Interaction between these two proteins detected by coimmunoprecipitation was quantified and plotted as ratio between the signal obtained from cells expressing both proteins, CRISP2-HA and PSP94-FLAG (b), and the background signal from cells lacking CRISP2-HA (a). Data represent mean ± SD of three independent determinations, and statistical significance is indicated: ∗*p* ≤ 0.05. *E*, calcium releases the inhibitory action of PSP94 on sterol export by CRISP2. Quadruple mutant cells (*pry1Δ pry2Δ say1Δ hem1Δ*) expressing either CRISP2 alone or together with PSP94 were cultivated in media containing the indicated concentrations of CaCl_2_ (0, 5, 10 mM) and labeled with [^14^C]cholesterol. Lipids were extracted from the cell pellet (P) and the culture supernatant (S), separated by TLC, and quantified. *F*, export of radiolabeled cholesteryl acetate is plotted as export index in the graph. Data correspond to means ± SD of three independent determinations, and statistical significance is indicated: ∗∗*p* ≤ 0.01. N/A, not applicable.

Given that the antifungal activity of PSP94 has been shown to depend on calcium concentrations (35), we tested whether calcium affects the interaction of PSP94 with CRISP2 and sterol binding by CRISP2. When tested by MST, both CRISP2 and PSP94 bind calcium, CRISP2 with high affinity (*K*_*d*_ 52 ± 0.9 nM), and PSP94 with low micromolar affinity (*K*_*d*_ 376 ± 1.7 μM) (Fig. 8*A*). However, in the presence of calcium, the CRISP2–PSP94 heteromeric complex fails to form, suggesting that complex formation between the two proteins *in vitro* is affected by the presence of calcium (Fig. 8*B*).

To test whether calcium also affects the formation of the heteromeric complex *in vivo*, we analyzed complex formation by coimmunoprecipitation. Cells expressing CRISP2-HA and PSP94-FLAG were cultivated in the absence or presence of calcium (5 and 10 mM CaCl_2_), CRISP2-HA was immunoprecipitated from the culture medium, and Western blots were probed for the presence of PSP94-FLAG. Complex formation between CRISP2 and PSP94 was observed in cells grown in the absence of calcium, whereas complex formation was reduced in cells grown in the presence of 5 mM calcium and could no longer be detected when cells were grown in 10 mM calcium. These results confirm that formation of the heteromeric complex is sensitive to high calcium concentrations (Fig. 8, *C* and *D*).

To examine whether disruption of complex formation between CRISP2 and PSP94 restores sterol binding and export by CRISP2 *in vivo*, we labeled cells with [^14^C]cholesterol and analyzed sterol export in cells grown in the presence of calcium. High calcium concentrations (10 mM) restored sterol binding and export by CRISP2 even in the presence of PSP94, indicating that PSP94 fails to inhibit sterol binding by CRISP2 in the presence of calcium (Fig. 8, *E* and *F*). Taken together, these results indicate that calcium modulates formation of the heteromeric complex between CRISP2 and PSP94 and thereby affects the sterol binding properties of CRISP2.

## Discussion

Here we focus on the interaction between the mammalian CAP superfamily member CRISP2 and the prostate secretory protein PSP94. We show that PSP94 modulates sterol binding by CRISP2 in a calcium-sensitive manner. In the absence of calcium, or at low calcium concentrations, PSP94 binds CRISP2 and other CAP family members, such as CRISP3 and Pry1, and blocks their propensity to bind sterols. At high calcium concentrations, on the other hand, PSP94 fails to bind CRISP2, which then can bind sterols even in the presence of PSP94. Modeling of the heteromeric complex reveals that binding of PSP94 to CRISP2 affects the hydrophobic loop harboring the CBM, which is important for sterol binding by the CAP domain, but the interaction does not affect the fatty acid–binding pocket of CRISP2 (6, 30, 31). Consistent with this, coexpression of PSP94 with CAP family proteins results in an inhibition of sterol export by CAP proteins but does not affect the export of fatty acids. Two conserved amino acids within the second N-terminal beta-strand of CRISP2 (L98 and Y99) were identified as being crucial for the interaction with PSP94. Mutations of these residues renders the sterol binding function of CRISP2 insensitive to inhibition by PSP94.

PSP94 is one of the major proteins secreted by the epithelial cells of the prostate and reaches a seminal plasma concentration of up to 1 mg/ml (16, 36). PSP94 inhibits growth of cancer cells in an experimental model of prostate cancer, but this property might be cell line specific (37, 38, 39, 40). Decreased expression of PSP94 is associated with an increased risk of developing prostate cancer, and its expression is reduced during cancer progression (41), suggesting that PSP94 has a protective function in cancer development. Expression levels of PSP94 might thus serve as a biomarker for prostate cancer (42).

A protective role of PSP94 might be explained by its antifungal activity, as fungal infection of the prostate can promote chronic prostatitis and infections by fungi such as *Malassezia* species may promote carcinogenesis (43, 44, 45, 46). *Malassezia* are lipophilic basidiomycetous yeasts that secrete extracellular lipases and take up lipids from their human and animal hosts (47, 48). Of interest, PSP94 has antifungal activity at low pH, but not at neutral pH in the presence of calcium (35). PSP94 may exert this activity by extracting the fungal-specific sterol, ergosterol, from the fungal plasma membrane. Consistent with such a possible mode of action, PSP94 disrupts liposomes that are rich in ergosterol but not those containing the mammalian cholesterol (35). This might explain the growth inhibition of PSP94 against fungal but not mammalian cells. Thus, fungal infections may be restricted when high levels of PSP94 are present but then start to proliferate and promote prostatitis when PSP94 levels decline.

Prostate cancer progression and mortality are associated with changes in calcium metabolism, parathyroid hormone levels, vitamin D levels, and androgen signaling (49, 50, 51). The observation that the expression of both CRISP2 and CRISP3 is increased in prostate cancer, whereas that of PSP94 is decreased, suggests that these two proteins could exert opposing functions during cancer progression (15, 42, 52). Both CRISP2 and PSP94 are involved in prostate cancer development and spermatogenesis, and lipids play crucial roles in both processes. For example, reduction in serum cholesterol levels, the precursor for sex steroid hormone synthesis, has been demonstrated to lower tumor androgen levels and slow tumor growth in xenograft models of human prostate cancer (53, 54, 55). In addition, calcium levels typically increase during prostate cancer progression (49), which favors cancer cell proliferation and metastasis (56, 57). On the other hand, high calcium levels could impair the protective role of PSP94, as its antifungal activity is inhibited by calcium (35). At the same time, high calcium concentrations disrupt the interaction between CRISP2 and PSP94 and thus release the sterol binding function of CRISPs. It seems possible that CRISP proteins could bind vitamin D_3_/cholecalciferol and thereby promote cancer development in the absence of PSP94 or at elevated calcium concentrations (50, 58). Thus, low levels of PSP94 could facilitate vitamin D3 binding by CRISP proteins and thus promote prostate cancer development and progression.

High-affinity, heteromeric complex formation between small secreted proteins and CAP family members is not limited to mammalian PSP94 and CRISP proteins but appears to be an evolutionary conserved mode of action to inhibit CRISP function. Venomous snakes, for example, neutralize the autotoxicity of their toxins through complex formation with endogenous proteins such as the small serum protein, SSP-2. SSP-2 belongs to the PSP94 family of proteins and interacts with triflin, a member of the CRISP family, which blocks smooth muscle contraction (59). The structural basis of this interaction was recently revealed by determining the crystal structure of the triflin–SSP2 heteromeric complex (60). SSP-2 binds the CAP domain of triflin and interacts with the CAP cavity, which results in tilting of the C-terminal CRD of triflin, thought to be responsible for its Ca^2+^-channel blocking activity (11, 60, 61). This mode of binding is likely conserved between the snake complex and human PSP94–CRISPs (13). Given that the interaction between PSP94 and CRISP2 is calcium sensitive, it seems possible that the SSP-2–triflin complex would dissociate upon entry into the bloodstream of the prey, thereby releasing the toxic action of triflin.

Taken together, the reported reciprocal regulation between CRISP and PSP94 levels during prostate cancer progression, the antifungal activity of PSP94, the sterol binding activity of CRISP proteins, and the calcium sensitivity of the interaction between CRISP and PSP94 suggest that a complex interplay between these activities is crucial for preventing malignant development of the prostate tissue. The observation that sterol binding of CRISP proteins is inhibited by PSP94 and modulated by calcium levels warrants further investigation into the physiological significance of this interaction in cancer development and progression.

## Experimental procedures

### *Saccharomyces cerevisiae* strains, growth conditions, and plasmids

*S. cerevisiae* double mutant *say1Δ hem1Δ* (*2Δ*) and quadruple mutant *say1Δ hem1Δ pry1Δ pry2Δ* (*4Δ*) strains were generated using PCR deletion cassettes and marker rescue strategies. Double mutant strains were grown in yeast extract, peptone, dextrose media, whereas quadruple mutants were grown in synthetic complete (SC) media. To compensate for heme deficiency, cells were cultivated in media supplemented with either delta-aminolevulinic acid (10 μg/ml) or cholesterol (20 μg/ml) dissolved in Tween-80.

Plasmids encoding for CAP proteins were constructed by cloning of PCR-amplified fragments from *S. cerevisiae* genomic DNA or codon-optimized synthesized genes (GenScript) into plasmid pRS416, containing an *URA3* selection marker. Genes were expressed from an *ADH1* promoter and fused to the signal sequence of alpha factor. Codon-optimized PSP94 was cloned into pRS414 using hygromycin as the selection marker. PSP94 was expressed from an *ADH1* promoter and fused to the signal sequence of Pry1.

### *In vivo* lipid export assay

The sterol export assay was performed as described (29). *S. cerevisiae* mutants deficient in heme biosynthesis (*hem1*Δ) and lacking the sterol deacetylase enzyme Say1 (*say1*Δ) were grown overnight in the presence of cold cholesterol/Tween-80. For strains containing pRS414, 80 μg/ml hygromycin was added to the medium. On the second day, cells were harvested by centrifugation, washed twice with SC medium, and then diluted to *A*_600nm_ of 1 into fresh medium containing 0.025 μCi/ml [^14^C]cholesterol. After overnight growth, cells were washed again with SC medium and cultivated for another day with nonradiolabeled cholesterol–containing medium. Cells were then centrifuged, and lipids were extracted from both the cell pellet and the culture supernatant using chloroform/methanol (1:1, v/v). Extracted radiolabeled lipids were quantified by scintillation counting, and volumes corresponding to 10,000 cpm (counts per minute) were dried. Dried lipids were resuspended in chloroform/methanol and separated by thin layer chromatography (TLC) on silica gel 60 plates (Merck) using the solvent system petroleum ether/diethyl ether/acetic acid (70:30:2, v/v/v). TLC plates were then exposed to phosphorimager screens, and radiolabeled lipids were visualized and quantified using a phosphorimager. The sterol export index was calculated as the ratio of extracellular cholesteryl acetate to the sum of intracellular and extracellular cholesteryl acetate. Export experiments were performed in triplicate, and the export index is given as mean ± SD of three independent experiments. Statistical analysis was performed with an unpaired *t* test using Prism (GraphPad Software).

### Protein secretion analysis and Western blotting

To analyze the expression and secretion of CAP proteins, they were tagged with a hemagglutinin (HA) tag (YPYDVPDYA) and PSP94 was tagged with a FLAG tag (DYKDDDDK). Epitope-tagged proteins were extracted from 3 *A*_600nm_ units of cells with 0.185 M NaOH (62), followed by precipitation with 10% TCA. In order to analyze their secretion into the culture medium, total proteins from 20 ml culture medium were precipitated with 10% TCA, acetone washed, solubilized in sample buffer, and analyzed by SDS-PAGE and Western blotting. Western blotting was performed using rat anti-HA antibody (rat, 1:2000, Roche #11867423001) and FLAG monoclonal antibody (mouse, 1:5000, Invitrogen #13-2500). As secondary antibodies: Goat Anti-Rat IgG antibody, Horseradish Peroxidase (HRP) conjugate (1:10000, Merck #AP136P), and Goat Anti-Mouse IgG (HRP) conjugates (1:10000, Bio-Rad #1706516) were employed. ECL Prime chemiluminescence substrate (Sigma-Aldrich) was used for signal development, and chemiluminescence was detected with an ImageQuant LAS 4000 biomolecular imager (GE Healthcare). Experiments were performed in triplicates with similar results.

### Protein expression and purification

DNA encoding proteins of interest were PCR amplified and cloned into NcoI and XhoI restriction sites of pET22b, which contains a PelB signal sequence to direct the secretion of expressed protein into the periplasmic space. Plasmids were transformed into *E. coli* BL21, and proteins were expressed with a C-terminal polyhistidine tag. Different induction strategies were used: overnight induction with lactose at 24 °C for Pry1, CRISP2, and PSP94 and 6 h of induction with 0.4 mM IPTG at 24 °C for the CAP domain of Pry1. Cells were collected and lysed, and the soluble cell lysate was incubated with nickel-nitrilotriacetic acid beads as per the manufacturer instructions (Qiagen). Beads were washed and loaded onto a Ni^2+^-NTA column, and proteins were eluted with a buffer containing 60 mM NaH_2_PO_4_, 300 mM NaCl, and 300 mM imidazole, pH 8.0. For MST experiments, proteins were applied to Zeba desalting spin columns (Thermo Scientific) and the buffer was exchanged to 60 mM NaH_2_PO_4_, 300 mM NaCl, pH 8.0. Protein concentration was determined by Lowry assay using the Folin reagent and bovine serum albumin as protein standard.

### Purification of CRISP3 under denaturing conditions

The CRISP3 gene was cloned into XhoI and NcoI restriction sites of pET22b vector. Protein expression was induced overnight with lactose at 24 °C. As CRISP3 aggregated in inclusion bodies, it was purified under denaturing conditions. After induction, cells were collected, lysed, and washed twice with 500 mM NaCl, 50 mM Tris, pH 8.5. The pellet was then resuspended with solubilization buffer (4% CHAPS, 7 M urea, 2 mM thiourea, 5% glycerol, 50 mM Tris HCl, pH 8.8), sonicated for 10 min, and incubated on shaker at 1300 rpm for 30 min at 55 °C. Urea was diluted to 4 M with binding buffer (300 mM NaCl, 60 mM NaH_2_PO_4_, pH 8.0). Affinity purification of the renatured protein was performed as described above.

### Microscale thermophoresis

MST experiments were performed to assess protein–protein, protein–lipid, and protein–calcium interactions using a Monolith NT.115 (Nanotemper Technologies). Proteins were labeled using the RED-tris-NTA His tag protein labeling kit. Labeled CAP proteins, 100 nM, were mixed with a serial dilution of unlabeled PSP94, PSP9^F24G^, cholesterol sulfate, or calcium chloride, prepared in binding buffer (20 mM Tris pH 7.5, 30 mM NaCl, 0.05% Triton X-100). Samples were loaded into MST standard capillaries, and MST measurements were performed using an 80% laser power setting. The dissociation constant *K*_*d*_ was obtained by plotting the normalized fluorescence (Fnorm) against the logarithm of ligand concentration. Experiments were performed in triplicates, and data were fitted using the *K*_*d*_ model of the MO.Affinity Analysis software (Nanotemper Technologies). For binding inhibition studies, CAP proteins and PSP94 were preincubated for 5 min at 24 °C before performing binding studies with cholesterol sulfate.

### Coimmunoprecipitation analysis

For Coimmunoprecipitation analysis, CRISP2 wildtype and mutant versions were tagged with an HA epitope tag, and wildtype PSP94 and the PSP94^F24G^ mutant version were fused to a FLAG tag. Yeast strains expressing these proteins were grown overnight and collected by centrifugation. The cell pellet was resuspended in lysis buffer (50 mM Tris-HCl pH 7.5, 100 mM NaCl, 0.1% NP-40, 10% glycerol, protease inhibitor, and 1 mM PMSF), and cells were broken with glass beads in a Precellys homogenizer. The culture medium was concentrated using size-exclusion protein concentrator spin columns (3K MWCO, Pierce, Thermo Scientific). Protein concentration was estimated by Bradford assay (Bio-Rad), and both the cell lysate and concentrated culture medium were incubated overnight at 4 °C with anti-HA magnetic beads (Pierce, Thermo Scientific). Beads were collected, washed with lysis buffer, and eluted by the addition of 2x reducing protein sample buffer. Samples were denatured for 10 min at 95 °C and analyzed by SDS-PAGE and Western blotting. Experiments were performed in triplicates with similar results.

### Fatty acid export

To analyze the CRISP2-dependent export of fatty acids, cells were grown overnight and the supernatant of 5 *A*_600nm_ units of yeast cells was collected and dried in a freeze dryer. Fatty acid methyl esters (FAMEs) were produced by incubating the dried fractions at 85 °C for 45 min in 1 ml of methanol–sulfuric acid (5% v/v) supplemented with butylated hydroxytoluene (0.01% w/v). The FAMEs were extracted in a mixture containing 1.5 ml of NaCl (0.9% w/v) and 2 ml of hexane, and the upper phase was collected. FAMEs were then resuspended in heptane and separated on an Agilent 7890A gas chromatograph equipped with a DB-23 capillary column (30 m × 0.25 mm × 0.25 μm) (Agilent Technologies) and quantified relative to an internal standard (C17:0, 10 μg) as described (31).

## Data availability

All data are contained within the article.

## Supporting information

This article contains supporting information.

## Conflict of interest

The authors declare that they have no conflict of interest with the content of this article.
