# Supplementary material for: Prostate secretory protein 94 inhibits sterol binding and export by the mammalian CAP protein CRISP2 in a calcium-sensitive manner
Source: J Biol Chem. 2022 Jan 19;298(3):101600. doi: 10.1016/j.jbc.2022.101600 (PMC8857485; doi:10.1016/j.jbc.2022.101600)
Supplement: Supplemental Figures S1 and S2 [file mmc1.pdf]

Supporting Information

Figure S1. PSP94 does not inhibit fatty acid binding of CRISP2 *in vivo*

Quadruple mutant cells lacking the acyl-CoA synthetases *FAA1* and *FAA4* and the yeast CAP family members *PRY1* and *PRY3* (*faa1*Δ *faa4*Δ *pry1*Δ *pry3*Δ) expressing different combinations of plasmid-borne copies of *PRY1*, *CRISP2,* and or *PSP94,* or their respective empty vectors (pRS423 or pRS426) were cultivated overnight. The export of fatty acids into the culture medium was then quantified. Data correspond to means ±S.D. of at least 3 independent experiments and statistical significance is indicated: **, $p < 0.01$, ***, $p < 0.001$ and ****, $p < 0.0001$.

Figure S2. Single point mutant versions of CRISP2 still interact with PSP94

(A-C) Each one of the three conserved amino acids in the β2-strand of CRISP2 that are predicted to establish interactions with PSP94 were mutated individually. The N97P, L98G and Y99P mutant versions of CRISP2 were expressed in *E. coli* and purified. Their interactions with PSP94 (panel A), and with cholesterol sulfate in the absence (panel B) or presence of PSP94 (panel C) was assessed by microscale thermophoresis. Measurements were performed in triplicates and the corresponding dissociation constants (K_d) are indicated. N/A; not applicable.

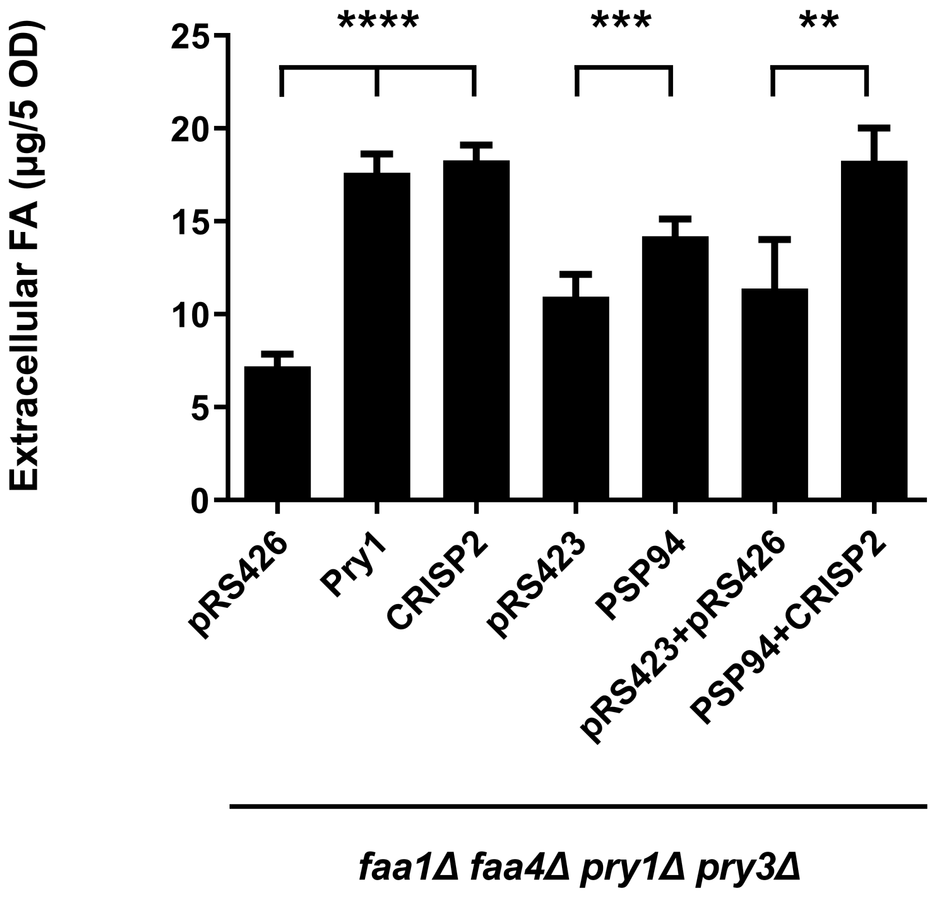
Extracellular FA (µg/5 OD)
25
20
15
10
5
0
****
***
**
pRS426
Pry1
CRISP2
pRS423
PSP94
pRS423+pRS426
PSP94+CRISP2
faa1Δ faa4Δ pry1Δ pry3Δ

Figure S1

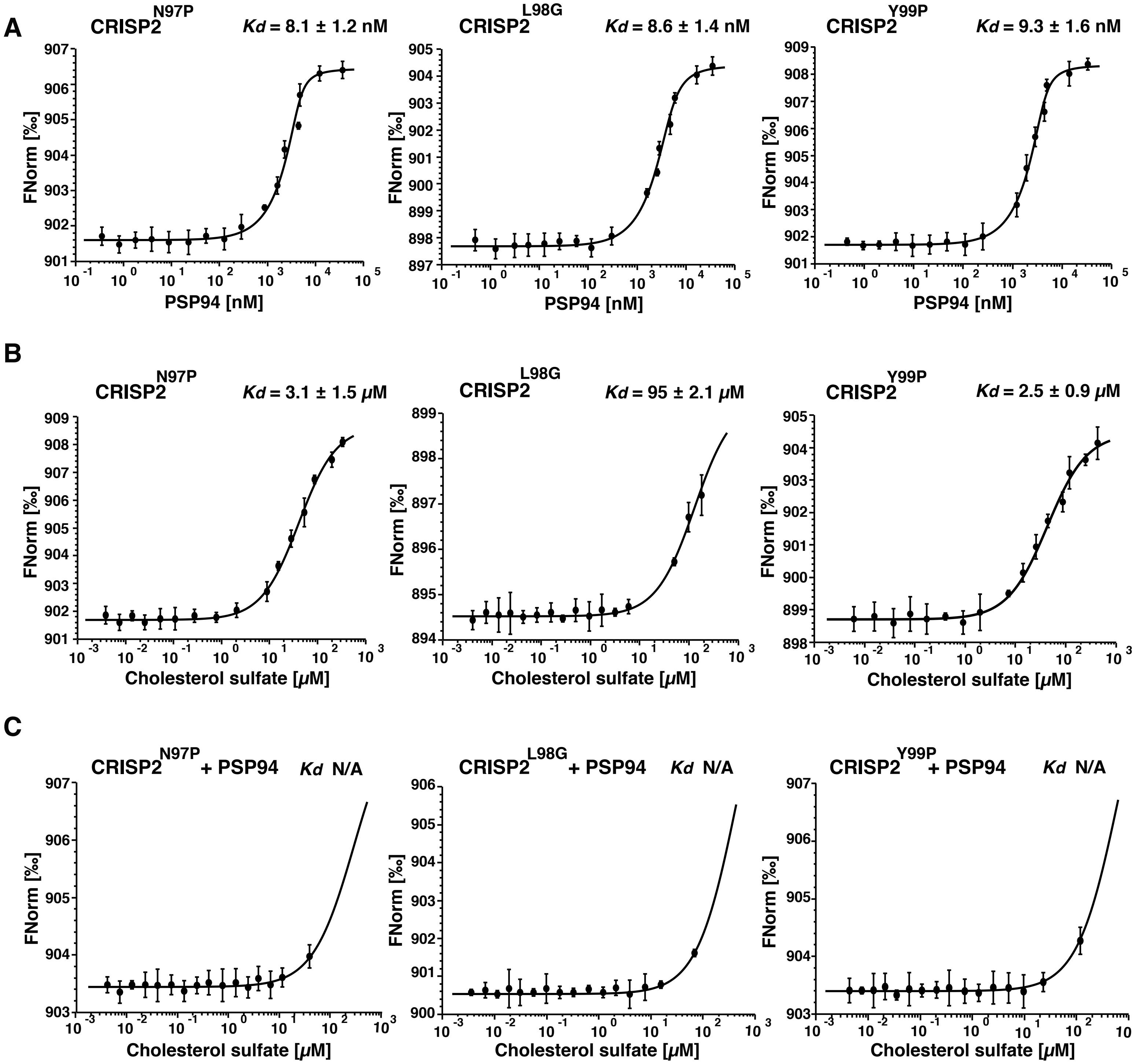

A
CRISP2 N97P Kd = 8.1 ± 1.2 nM
CRISP2 L98G Kd = 8.6 ± 1.4 nM
CRISP2 Y99P Kd = 9.3 ± 1.6 nM
FNorm [‰]
PSP94 [nM]
B
CRISP2 N97P Kd = 3.1 ± 1.5 μM
CRISP2 L98G Kd = 95 ± 2.1 μM
CRISP2 Y99P Kd = 2.5 ± 0.9 μM
FNorm [‰]
Cholesterol sulfate [μM]
C
CRISP2 N97P + PSP94 Kd N/A
CRISP2 L98G + PSP94 Kd N/A
CRISP2 Y99P + PSP94 Kd N/A
FNorm [‰]
Cholesterol sulfate [μM]

Figure S2
